# Physicians’ Confidence in Primary Palliative Care and Preferred Methods of Responding: A Sequential Mixed-Methods Survey

**DOI:** 10.1089/pmr.2024.0110

**Published:** 2025-04-29

**Authors:** Rachel D. Havyer, Rachel M. Wiste, Cory Ingram, Jennifer L. Ridgeway, Kathleen J. Yost

**Affiliations:** ^1^ Division of Community Internal Medicine, Geriatrics, and Palliative Care, Mayo Clinic, Rochester, Minnesota, USA.; ^2^ Robert D. and Patricia E. Kern Center for the Science of Health Care Delivery, Mayo Clinic, Rochester, Minnesota, USA.; ^3^ Department of Quantitative Health Sciences, Mayo Clinic, Rochester, Minnesota, USA.

**Keywords:** generalist palliative care, specialty palliative care, utilization

## Abstract

**Background:**

Collaborative methods are necessary to meet patient palliative care (PC) needs because of the inadequate supply of PC specialists.

**Objective:**

This study aimed to conduct a needs assessment and determine primary care, emergency, and hospital physicians’ general attitudes about primary PCs, confidence in managing common PC scenarios, and preferences for interaction with specialty PCs.

**Design:**

A sequential mixed-methods study design was used, whereby individual qualitative interviews informed the content of a quantitative survey. Semistructured telephone interviews were conducted by a member of the study team with expertise in qualitative research methods.

**Setting/Subjects:**

The quantitative survey, delivered to primary care, emergency, and hospital physicians across four distinct geographic locations of a large health system, solicited impressions on common clinical PC scenarios that might pose challenges.

**Measurements:**

Survey data included demographic information, clinician confidence levels, preferences for support in managing PC scenarios, and likelihood to refer to PC.

**Results:**

The quantitative survey was completed by 126 physicians (response rate, 13.9%). Overall mean (standard deviation) confidence levels were lowest for a scenario about handling pain (5.57 [2.35] out of 10) and highest for goals-of-care conversations with the patient (7.80 [2.02]). Spearman correlations between mean confidence and likelihood to refer to PC demonstrated weak to moderate inverse correlations. Respondents with previous training in PC had higher mean confidence in managing symptoms and goals-of-care conversations.

**Conclusions:**

Continuing efforts are needed to help improve physicians’ confidence in primary PC skills and develop innovative methods to provide collaborative support of specialty PCs across various specialties and PC needs.

## Background

The involvement of palliative care (PC) among critically ill patients leads to better symptom control, improved quality of life, and reduced costs,^1,2^ and thus the demand for PC is increasing.^3^ Current and projected shortages of specialty-trained PC clinicians and increasing PC needs of the aging population suggest the need for new models of PC delivery, such as the integration of primary palliative care (PPC) and specialty palliative care (SPC) into frontline practice.^4,5^

In a coordinated PC model, the frontline clinician manages PPC needs, including initial discussions of goals of care, code status, and basic symptom management. Coordinated and integrated SPC resources are involved when patient complexity overwhelms the level of comfort of the frontline clinician.^5^ Many barriers to effectively delivering PPC have been reported in the literature, however, including clinicians’ lack of skills and confidence, bureaucratic procedures, care collaboration between professionals, and communication with patients about prognosis and treatment decision making.^6,7^ Furthermore, a study integrating the views of medical professionals with those of patients and family indicated that key aspects of PPC delivery include improving both knowledge and skills in PPC and the coordination of PC delivery.^8^

As a first step to developing a collaborative model in our health care system, we aimed to conduct a needs assessment to better understand the practice, clinician, and patient factors that would lead clinicians to refer to SPC. This assessment would be used to identify areas of PPC in which physicians may need educational intervention or improved access to collaboration with SPC. We assessed physician attitudes about PPC, confidence in managing common PC patient scenarios, and preferences for the method of collaboration with SPC. The needs assessment was conducted across our diverse health care system, which includes academic tertiary care centers with access to inpatient and outpatient SPC resources, as well as rural clinics and hospitals with hospice but no other integrated SPC services, to gather a complete picture of current state and preferred methods of collaboration.

## Methods

This needs assessment study was deemed by the Mayo Clinic Institutional Review Board (IRB #16-005229) to be not “human subjects research.” A sequential mixed-methods study design was used, whereby qualitative individual interviews informed the content of a quantitative survey.^9^

### Qualitative interviews

Semistructured telephone interviews were conducted with primary care physicians and hospitalists from August 8 to September 7, 2016, by a member of the study team with expertise in qualitative research methods (J.L.R.). Physicians were purposely sampled from all 3 main campuses of Mayo Clinic (Rochester, Minnesota; Arizona; and Florida) and 4 locations of the Mayo Clinic Health System, which serves rural communities in southern Minnesota and western Wisconsin. Topics in the semistructured interview guide included responsibilities related to providing PPC, confidence in providing PPC, barriers to providing PPC, history of referral to SPC, reasons for and barriers preventing referral to SPC, resources that would enhance the provision of PPC, preferences for mode of interacting with SPC clinicians, and availability and/or need for PC services in the community. Interviews were audio recorded, and detailed notes from each interview were reviewed by the multidisciplinary members of the study team to determine the ideal content and format of quantitative survey questions. No identifiable or protected health information was collected in the qualitative interviews or quantitative web surveys.

### Quantitative surveys

#### Content

Assessment of the qualitative interviews suggested that the ideal format for standardizing questions for the quantitative survey would be to present vignettes of common clinical scenarios that might challenge a frontline clinician from a PC perspective. Definitions of PPC and SPC were given to ensure consistent interpretation of terms.

The survey was developed by the authors based on qualitative interviews (Supplementary Data). This anonymous survey was designed in Qualtrics by the Mayo Clinic Survey Research Center. Respondents were asked to report their specialty, years in practice, awareness of SPC, formal training in PC (described as PC fellowship, dedicated PC continuing medical education, or training activities), desire for more training, and estimated proportion of their patients in need of PC. To manage respondent burden, we limited the survey to six vignettes reflecting common scenarios (Supplementary Appendix): (1) managing family dynamics, (2) managing refractory symptoms, (3) managing pain, (4) managing emotional and spiritual distress, (5) goals-of-care conversation with the patient, and (6) goals-of-care conversation with spouse (or surrogate decision maker). For each vignette, respondents rated their confidence in managing the PC needs themselves for that scenario on a numeric rating scale of 0, *not at all confident*, to 10, *completely confident*. For each vignette, they also indicated from which one or more resources they would request help (i.e., SPC, hospice, social work, chaplain, oncology, volunteers trained in advance care planning, psychiatry, ethics consult, gastroenterology, surgery, other, or none), with instructions to respond as if all resources were currently accessible in their practice.

Respondents were asked how likely they would be to request input from SPC (not at all likely, a little bit likely, somewhat likely, or very likely) for each vignette. They were asked to indicate their preference for interactions with SPC, specifically the type of interaction that would be most helpful for each specific vignette, with the following options: an integrated community specialist (ICS),^10–12^ which could include primary/specialty collaboration with options of collocated visits, e-consults, or curbsides; a telephone conversation with SPC; reviewing SPC notes in the electronic health record (EHR); curbside consult (e.g., calling a known colleague for advice); e-consult (SPC reviews the case electronically and provides recommendations on management in a dedicated note in the EHR); a traveling specialist; virtual consult; AskMayoExpert: Care Process Model (an institutional database offering access to standardized practice recommendations on various medical conditions)^13^; email interaction; or other methods of interaction.

#### Administration

Qualtrics web software was used to administer the survey in May and June 2018. All physicians in all geographic areas of our institution with a primary affiliation in three clinical areas (primary care internal medicine/family medicine, emergency department [ED], and hospital internal medicine) were sent an email invitation with a link to the web-based survey. Two reminder emails with the survey link were sent.

### Analysis

Survey data were reported with descriptive statistics. We reviewed respondent-reported characteristics to assess whether practice and physician factors influenced responses to the standardized vignettes (practice settings, estimates of patients needing PC, and prior training in PC). We calculated mean confidence ratings for each vignette and for the overall mean confidence across all six vignettes. Associations between overall mean confidence rating and provider type (primary care, ED, hospital medicine) were assessed with analysis of variance, and associations between overall mean confidence and prior training in PC were assessed with the independent-sample *t* test. Spearman correlations were calculated for correlations between confidence level and likelihood to refer to SPC. Statistical analysis was performed with SAS version 9.4 (SAS Institute Inc). *p* < 0.05 was considered statistically significant.

## Results

Six primary care physicians and two hospitalists completed the qualitative interviews (duration range, 11–22 minutes). After the development of the web-based quantitative survey, 906 physicians were sent an email invitation with a link to the survey. The survey was completed by 126 of 906 invited physicians (response rate, 13.9%). Demographic characteristics of the respondents are shown in Table 1. Most physicians practiced in primary care (80/126; 63.5%), followed by hospital medicine (30/126; 23.8%) and emergency medicine (16/126; 12.7%). Physicians had been in practice for a mean of 16.8 years (range, 0–40 years) and estimated that a mean of 21.8% (range, 2 − 100%) of the patients they treated needed PC. The majority of physicians (83, 65.8%) were aware of the existence of the SPC consulting service before the survey, and the vast majority of these physicians (70/83, 84%) had used the SPC consulting service previously. Only 31 respondents (24.6%) reported having formal training in PC, 74% of whom (*n* = 23) indicated that they would like additional training in PC. Of the 72 respondents who indicated they had no formal training in PC, 49 (68%) indicated that they would like to receive training in PC.

**Table 1.** Characteristics of Survey Respondents

Characteristic	Value^a^ (*N* = 126)
Physician specialty	
Emergency department	16 (12.7)
Hospitalist	30 (23.8)
Primary care	80 (63.5)
Years in practice, mean (SD)	16.8 (11.0)
Awareness of palliative care consulting service before survey	
Yes	83 (65.8)
No	19 (15.1)
Missing	24 (19.0)
Formal training in palliative care	
Yes	31 (24.6)
No	72 (57.1)
Missing	23 (18.3)
Estimated proportion of respondent’s patients needing palliative care, mean (range)	21.8% (2 − 100%)

^a^ Values are no. (%) of respondents unless otherwise stated.

SD, standard deviation.

**FIG. 1.**
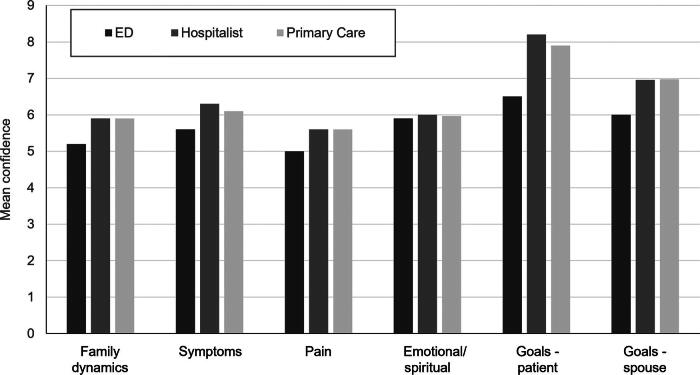
Physician confidence in palliative care topics by specialty. Graph reports mean confidence levels (out of 10) in six vignette topics for emergency department (ED), hospitalist, and primary care physicians.

Physician ratings of their confidence in handling several common PC clinical scenarios based on the six vignettes are summarized in Table 2 and separated by specialty in Figure 1. Mean confidence levels across all specialties were lowest for the vignette on handling pain and highest for goals-of-care conversations with the patient (Table 2). Overall mean confidence across all vignettes was higher for respondents with (*n* = 31) versus without (*n* = 72) prior PC training (7.1 vs. 6.1; *p* = 0.009). No significant differences in confidence levels across physicians in different specialties were noted within each vignette (all *p* > 0.05) or for the mean confidence when averaged across all six vignettes (*p* = 0.24). Pearson correlations between years in clinical practice and levels of confidence for any individual vignette or for overall confidence across vignettes were not statistically significant (All *r* < 0.1, all *p* > 0.05).

**Table 2.** Physician Confidence in Independently Managing Clinical Scenarios

Vignette	*N*	Mean (SD) confidence level^a^
Family dynamics	126	5.81 (2.46)
Symptoms	114	6.09 (2.39)
Pain	111	5.57 (2.35)
Emotional and spiritual distress	106	5.97 (2.30)
Goals-of-care conversation with patient	104	7.80 (2.02)
Goals-of-care conversation with spouse	102	6.88 (2.12)

^a^ Confidence level measured on a scale of 0 (not at all confident) to 10 (completely confident).

**FIG. 2.**
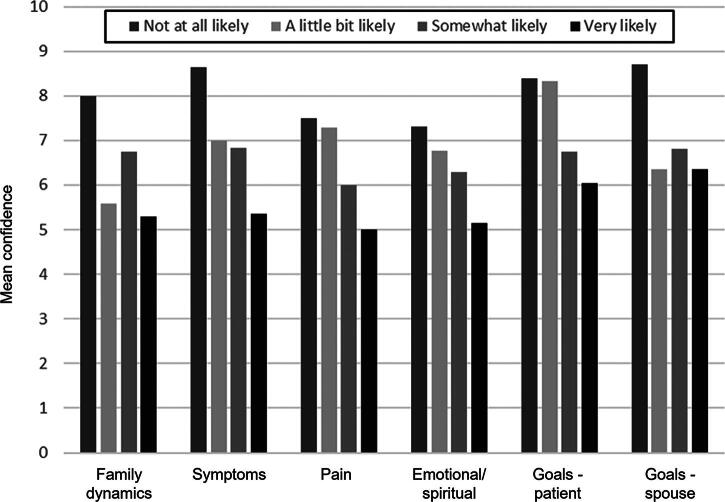
Physician confidence in palliative care topics by likelihood to request SPC input. Graph reports mean confidence levels (out of 10) in six vignette topics for physicians by their reported likelihood of requesting input from SPC. SPC indicates specialty palliative care.

Figure 2 presents a mean respondent level of confidence in each vignette based on their likelihood of consulting SPC. Spearman correlations between mean confidence level and the likelihood to request SPC demonstrated weak to moderate inverse correlations for each vignette as follows: family dynamics –0.28 (*p* = 0.002); symptoms –0.41 (*p* < 0.001); pain −0.33 (*p* < 0.001); emotional/spiritual distress –0.33 (*p* < 0.001); discussing goals of care with the patient –0.43 (*p* < 0.001); and discussing goals of care with spouse –0.31 (*p* = 0.001).

Respondents selected various resources from which they could request help for each vignette (could select all that applied for each) (Table 3). Across all vignettes except for goals-of-care conversation with the patient, the most common resource from which respondents would request help was SPC. For the vignette on goals-of-care conversation with the patient, the respondents would most commonly request help from a social worker or volunteer trained in advance care planning; in this vignette, compared with other vignettes, respondents also most commonly indicated that they would not request help from any outside resources.

**Table 3.** Number of Respondents Who Would Request a Resource or Specialty to Help Manage Each Scenario

	Vignette					
Resource/service	Family dynamics	Symptoms	Pain	Emotional/spiritual	Goals—patient	Goals—spouse
Specialty palliative care	97	92	97	79	28	73
Hospice	58	79	60	37	10	28
Social work	81	20	7	45	47	46
Chaplain	70	17	10	59	14	47
Oncology	NA	43	45	NA	NA	NA
Volunteers trained in advance care planning	NA	NA	NA	22	44	19
Psychiatry	15	3	5	53	5	2
Ethics consult	47	NA	NA	14	2	5
Gastroenterology	NA	34	NA	NA	NA	NA
Surgery	NA	11	5	0	NA	NA
Other	7	4	17	4	3	7
None	1	2	2	5	25	7

NA, not applicable to the vignette.

Respondents indicated their preferred methods for interacting with SPC when requested for each vignette (Table 4). Of the options for interaction, the most selected methods of assistance across all vignettes were ICS, telephone conversation, or reviewing SPC notes in the EHR. The top three preferences for method of SPC interaction for each vignette were reviewing SPC notes, telephone conversation, and ICS for family dynamics; telephone conversation, ICS, and e-consult for symptom management; ICS, telephone, and reviewing SPC notes for pain; ICS, telephone, and reviewing SPC notes/curbside consult for emotional/spiritual; ICS, reviewing SPC notes, and telephone for goals with patient; and ICS, telephone, and reviewing SPC notes for goals with surrogate. Email was the least preferred selection for SPC interaction. There was a significant positive correlation between physicians’ estimates of the percentage of patients in need of PC and overall confidence across all vignettes (*r* = 0.29, *p* = 0.003).

**Table 4.** Preferred Methods of Interaction with Specialty Palliative Care

	Vignette^a^						Total across vignettes
Method of interaction	Family dynamics	Symptom	Pain	Emotional/spiritual	Goals—patient	Goals—spouse	
Integrated community specialist (e.g., primary/specialty collaboration, with options of collocated visits, e-consults, or curbsides)	39	47	57	49	23	42	257
Telephone conversation	40	52	50	40	18	30	230
Reviewing specialty palliative care notes in the electronic health record	79	32	35	25	19	25	215
Curbside (e.g., call a known colleague for advice)	17	30	32	25	11	11	126
E-consult (specialist clinician reviews case electronically and provides recommendations)	15	33	30	19	12	13	122
Traveling specialist	13	16	16	17	8	16	86
Virtual consults	6	17	14	14	7	11	69
Care process model	5	16	12	6	6	2	47
Email	3	11	7	7	3	2	33
Other	8	13	15	18	5	16	75

^a^ No. of respondents who chose each method of interaction with specialty palliative care, among those who indicated that method as a resource they would use to help manage each scenario. Respondents could select more than one option.

## Discussion

To develop an improved collaborative model of PPC and SPC delivery at our institution, a needs assessment was completed to ascertain the needs and preferences of frontline physicians in delivering PPC and collaborating with SPC. This study presents the perspective of frontline physicians in primary care, hospital, and emergency medicine who indicate a desire for assistance from SPC when presented with commonly encountered PC vignettes. The findings confirm the need to continue to maintain or improve access to SPC. Other interdisciplinary resources like social workers, chaplains, and advance care planning volunteers have commonly desired resources for support with non-symptom-based scenarios and may be additional means of extending PC support in areas without access to SPC. Respondents stated they would be less likely to request SPC help with goals-of-care conversations with the patient. It is possible that they have more experience with this skill because it is a basic skill of PPC^4^ and is taught in residency programs.^14^

The mean confidence levels of physicians to independently manage the scenarios were highest for goals-of-care conversations with the patient and lowest for managing pain symptoms. Across each vignette, the likelihood of requesting SPC assistance decreased with increasing levels of confidence. Additionally, respondents indicating prior PC training reported higher confidence. This finding is similar to that in a survey of primary care providers, which showed significant positive correlations with physician confidence in advance care planning discussions and their likelihood of initiating such discussions.^15^ Furthermore, a study of graduate medical trainees showed that exposure to PPC education was associated with higher confidence and increased frequency of implementing PPC skills.^16^ Therefore, continuing to expand efforts to train and build physicians’ confidence and skills in PPC at our institution, particularly in areas of symptom control, may help meet patients’ PPC needs when SPC resources are limited. Additionally, although the majority of physicians were aware of SPC resources, approximately one-third were unaware of the SPC service. Therefore, further efforts to raise awareness of SPC resources may help meet patients’ complex PC needs.

Although we did not observe a significant difference in confidence based on the specialty of the provider, ED physicians had slightly lower reported mean confidence levels in each vignette and in the overall mean confidence across vignettes. A review of Accreditation Council for Graduate Medical Education milestones across specialties for content relevant to PPC reported an increased number of salient PPC milestones in internal medicine and family medicine compared with emergency medicine.^17^ Thus, it is possible that an emergency medicine residency contains less exposure to PPC concepts than do the other residencies.

This study was novel in assessing physicians’ preferred methods of obtaining assistance to meet patients’ PC needs. Physician respondents were most likely to request help from SPC compared with other resources to meet most PC needs of their patients. Therefore, it is important to use innovative methods of collaborating with primary care physicians to meet this demand for SPC even amid a shortage of SPC-trained clinicians.^18^ Respondents indicated that they are amenable to various ways of interacting with SPC. The use of methods such as telephone conversations, e-consults, or virtual consults may allow a greater reach of the limited SPC resources to more patients. Furthermore, helping to preidentify and link the specific type of patient PC need to a more targeted resource in the interdisciplinary team (e.g., advance care planning, goals of care, or symptom management) may help improve efficiency and collaboration with primary teams. Continued research is needed to improve SPC collaboration and integration and reach with those providing PPC to best meet patient needs.

Limitations of this study include that the study period was before the COVID-19 pandemic, so the selection of virtual options for connection to patients and colleagues may be underrepresented in the preferences of respondents compared with current usage. Age and gender of respondents were not collected, so we are unable to determine whether responses vary by these demographics. In addition, the survey response rate was only 13.9%, which may have affected our ability to comprehensively describe the attitudes about PPC at our institution. Furthermore, the low response rate plus single institution setting may also limit the generalizability of our key findings. Although we chose vignettes to account for some of the most common clinical scenarios encountered in PPC, the vignettes may not be generalizable to all aspects of that specific topic, so confidence levels in topic areas must be interpreted with caution. Nevertheless, the findings indicate variation in confidence by scenarios, which may help inform broad areas to target for PPC training and SPC support. Additionally, this study was conducted within one medical institution, so the findings may not generalize to other institutions. However, our institution encompasses three geographically distinct areas in the United States and includes academic, nonacademic, and rural practices with a large variability of SPC resources and services. Although respondents were instructed to respond as if the resources were available, the lack of exposure to or experience with certain resources may have influenced the likelihood of choosing that resource.

In conclusion, primary care, hospital, and emergency medicine physicians indicate a strong desire to use an SPC team to help meet the needs of their patients. It is important to continue efforts to train physicians in PPC skills to help increase their confidence in meeting the PC needs of their patients. These data serve to highlight areas for the development of educational interventions in PPC and new methods of collaboration between PPC and SPC in our institution. For patients and clinicians requiring access to SPC resources, further use of and research on innovative methods of delivering SPC is needed across various specialties and PC needs.

## Data Statement

All relevant data supporting the findings of this study are reported within the article or are available from the corresponding author upon reasonable request.

## Abbreviations Used

ED: emergency department
EHR: electronic health record
ICS: integrated community specialist
PC: palliative care
PPC: primary palliative care
SPC: specialty palliative care
