# Supplementary material for: Physicians’ Confidence in Primary Palliative Care and Preferred Methods of Responding: A Sequential Mixed-Methods Survey
Source: Palliat Med Rep. 2025 Apr 29;6(1):215–22. doi: 10.1089/pmr.2024.0110 (PMC12410319; doi:10.1089/pmr.2024.0110)
Supplement: Supplementary Data [file pmr.2024.0110_supplementarydata.docx]

Primary Palliative Care

We are interested in understanding resources you might use when managing challenging scenarios that you could encounter in routine practice.

In this survey, Palliative Care refers to pain and symptom management, emotional and spiritual support, and advance care planning conversations when patients face a chronic, debilitating or life-threatening illness.  
 
We distinguish Primary Palliative Care from Specialty Palliative Care as follows:
 Primary Palliative Care is the basic provision of palliative care by clinicians, including primary care providers, hospitalists, specialists, emergency room clinicians, social workers, or others who are not working in Specialty Palliative Care.

Specialty Palliative Care is a service of dedicated palliative care specialists who are available for enterprise-wide consultations by contacting the Palliative Care Consulting Service.

There are six (6) scenarios in this survey.  Please read each scenario below and answer the questions.  For all questions, assume that the resources and services are currently available in your care setting.

|  |
| --- |
|  |

Q1 **Managing Family Dynamics**

*Mrs. Smith is an 83-year-old woman who has been hospitalized for 25 days following the development of a neurological condition known as posterior reversible encephalopathy syndrome. She is comatose and the consensus of the primary and neurology consulting teams is that she will not improve enough to survive this hospitalization.   She does not have an advance directive and her daughters are in conflict as to what their mother’s wishes would be. On multiple occasions, when you talk with the daughters, the conversations are very tense and do not reach consensus.*

 **On a scale of 0 to 10, where 0 is "Not at all confident" and 10 is "Completely confident", how confident are you in your ability to manage the family dynamics on your own?**

- Not at all confident 0 (0)
- 1 (1)
- 2 (2)
- 3 (3)
- 4 (4)
- 5 (5)
- 6 (6)
- 7 (7)
- 8 (8)
- 9 (9)
- Completely confident 10 (10)

Q2 **From which of the following resources or specialties would you request help to manage the family dynamics in this scenario?**

*Assume all of the resources/specialties are available to you.*
(Mark all that apply.)

- None (1)
- Chaplain (2)
- Ethics consult (3)
- Hospice (4)
- Psychiatry (5)
- Social worker (6)
- Specialty Palliative Care (7)
- Other (8) ________________________________________________

Q3 Assuming Specialty Palliative Care is available in your setting...
 **How likely is it that you would request input from Specialty Palliative Care to help manage the family dynamics in this scenario?**

- Not at all likely (1)
- A little bit likely (2)
- Somewhat likely (3)
- Very likely (4)

Q4 **How would you prefer to interact with Specialty Palliative Care in this scenario?**
 (Mark all that apply.)

- Care Process Model (1)
- Telephone conversation (2)
- E-consult (specialist provider reviews the case electronically and provides recommendations on management in a dedicated note in the electronic record) (3)
- Traveling Specialist (4)
- Integrated Community Specialist (e.g. primary/specialty collaboration with options of collocated visits, e-consults or curbsides) (5)
- Virtual consults (6)
- Curbside (e.g., call a known colleague for advice) (7)
- Email (8)
- Reviewing Specialty Palliative Care notes in the EHR (9)
- Other (10) ________________________________________________

|  |
| --- |

|  |
| --- |

Q5 **Managing Symptoms**

*Mr. Jones is a 54-year-old gentleman with widely metastatic rectal carcinoma with diffuse peritoneal cancer involvement.  He is losing his ability to maintain his oral hydration and nutrition, and his nausea has been refractory to the medications on the nausea order set.  He and his wife are struggling with how to proceed and are hoping his nausea can improve.*

 **On a scale of 0 to 10, where 0 is "Not at all confident" and 10 is "Completely confident", how confident are you in your ability to manage the patient's symptoms on your own?**

- Not at all confident 0 (0)
- 1 (1)
- 2 (2)
- 3 (3)
- 4 (4)
- 5 (5)
- 6 (6)
- 7 (7)
- 8 (8)
- 9 (9)
- Completely confident 10 (10)

Q6 **From which of the following resources or specialties would you request help to manage the patient's symptoms in this scenario?**

*Assume all of the resources/specialties are available to you.*
(Mark all that apply.)

- ⊗None (1)
- Chaplain (2)
- GI (3)
- Hospice (4)
- Oncology (5)
- Psychiatry (6)
- Social worker (7)
- Specialty Palliative Care (8)
- Surgery (9)
- Other (10) ________________________________________________

Q7 Assuming Specialty Palliative Care is available in your setting...
 **How likely is it that you would request input from Specialty Palliative Care to help manage the patient's symptoms in this scenario?**

- Not at all likely (1)
- A little bit likely (2)
- Somewhat likely (3)
- Very likely (4)

Q8 **How would you prefer to interact with Specialty Palliative Care in this scenario?**
 (Mark all that apply.)

- Care Process Model (1)
- Telephone conversation (2)
- E-consult (specialist provider reviews the case electronically and provides recommendations on management in a dedicated note in the electronic record) (3)
- Traveling Specialist (4)
- Integrated Community Specialist (e.g. primary/specialty collaboration with options of collocated visits, e-consults or curbsides) (5)
- Virtual consults (6)
- Curbside (e.g., call a known colleague for advice) (7)
- Email (8)
- Reviewing Specialty Palliative Care notes in the EHR (9)
- Other (10) ________________________________________________

|  |
| --- |

Q9 **Managing Pain**
    *Mr. Dolor is a 72-year-old man with metastatic prostate cancer who presents with persistent and severe pain that interferes with his function despite escalating doses of opioid pain medications.  A recent consultation with interventional pain colleagues resulted in interventions providing only temporary relief.  You are hoping to manage his pain medically.*

 **On a scale of 0 to 10, where 0 is "Not at all confident" and 10 is "Completely confident", how confident are you in your ability to manage the patient's pain on your own?**

- Not at all confident 0 (0)
- 1 (1)
- 2 (2)
- 3 (3)
- 4 (4)
- 5 (5)
- 6 (6)
- 7 (7)
- 8 (8)
- 9 (9)
- Completely confident 10 (10)

Q10 **From which of the following resources or specialties would you request help to manage the patient's pain in this scenario?**

*Assume all of the resources/specialties are available to you.*
(Mark all that apply.)

- ⊗None (1)
- Chaplain (2)
- Hospice (3)
- Oncology (4)
- Psychiatry (5)
- Social worker (6)
- Specialty Palliative Care (7)
- Surgery (8)
- Other (9) ________________________________________________

Q11 *Assuming Specialty Palliative Care is available in your setting...*
 **How likely is it that you would request input from Specialty Palliative Care to help manage the patient's pain in this scenario?**

- Not at all likely (1)
- A little bit likely (2)
- Somewhat likely (3)
- Very likely (4)

Q12 **How would you prefer to interact with Specialty Palliative Care in this scenario?**
 (Mark all that apply.)

- Care Process Model (1)
- Telephone conversation (2)
- E-consult (specialist provider reviews the case electronically and provides recommendations on management in a dedicated note in the electronic record) (3)
- Traveling Specialist (4)
- Integrated Community Specialist (e.g. primary/specialty collaboration with options of collocated visits, e-consults or curbsides) (5)
- Virtual consults (6)
- Curbside (e.g., call a known colleague for advice) (7)
- Email (8)
- Reviewing Specialty Palliative Care notes in the EHR (9)
- Other (10) ________________________________________________

|  |
| --- |

|  |
| --- |

Q13 **Managing Emotional and Spiritual Distress**
   *Mr. Pickle is a 42-year-old gentleman with advanced heart failure who has had repeated and prolonged hospitalizations.  On morning rounds, he expresses his wish to have his life ended.  He expresses ongoing frustration with his poor quality of life and just wants to ‘get this over with’. Mr. Pickle is very sick, however, not necessarily dying.  He is in extreme emotional distress.*

 **On a scale of 0 to 10, where 0 is "Not at all confident" and 10 is "Completely confident", how confident are you in your ability to manage the patient's distress on your own?**

- Not at all confident 0 (0)
- 1 (1)
- 2 (2)
- 3 (3)
- 4 (4)
- 5 (5)
- 6 (6)
- 7 (7)
- 8 (8)
- 9 (9)
- Completely confident 10 (10)

Q14 **From which of the following resources or specialties would you request help to manage the patient's distress in this scenario?**

*Assume all of the resources/specialties are available to you.*
(Mark all that apply.)

- ⊗None (1)
- Volunteers trained in Advanced Care Planning (2)
- Chaplain (3)
- Ethics consult (4)
- Hospice (5)
- Psychiatry (6)
- Social worker (7)
- Specialty Palliative Care (8)
- Surgery (9)
- Other (10) ________________________________________________

Q15 *Assuming Specialty Palliative Care is available in your setting...*

 **How likely is it that you would request input from Specialty Palliative Care to help manage the patient's distress in this scenario?**

- Not at all likely (1)
- A little bit likely (2)
- Somewhat likely (3)
- Very likely (4)

Q16 **How would you prefer to interact with Specialty Palliative Care in this scenario?**
 (Mark all that apply.)

- Care Process Model (1)
- Telephone conversation (2)
- E-consult (specialist provider reviews the case electronically and provides recommendations on management in a dedicated note in the electronic record) (3)
- Traveling Specialist (4)
- Integrated Community Specialist (e.g. primary/specialty collaboration with options of collocated visits, e-consults or curbsides) (5)
- Virtual consults (6)
- Curbside (e.g., call a known colleague for advice) (7)
- Email (8)
- Reviewing Specialty Palliative Care notes in the EHR (9)
- Other (10) ________________________________________________

|  |
| --- |

Q17 **Managing Goals of Care**
  Mrs. Anderson is an 83-year-old woman who you have been caring for over the course of many years but has begun to show the signs of mild cognitive impairment.  She has been quite independent over the years and has had strong opinions regarding her medical care.  At present she retains the capacity for decision making and expressing her goals.  You note that a conversation on advanced-care planning and goals of care is needed prior to her losing her ability to express her desires.

 **On a scale of 0 to 10, where 0 is "Not at all confident" and 10 is "Completely confident", how confident are you in your ability to manage the patient's goals of care on your own?**

- Not at all confident 0 (0)
- 1 (1)
- 2 (2)
- 3 (3)
- 4 (4)
- 5 (5)
- 6 (6)
- 7 (7)
- 8 (8)
- 9 (9)
- Completely confident 10 (10)

Q18 **From which of the following resources or specialties would you request help to manage the patient's goals of care in this scenario?**

*Assume all of the resources/specialties are available to you.*
(Mark all that apply.)

- ⊗None (1)
- Volunteers trained in Advanced Care Planning (2)
- Chaplain (3)
- Ethics consult (4)
- Hospice (5)
- Psychiatry (6)
- Social worker (7)
- Specialty Palliative Care (8)
- Other (9) ________________________________________________

Q19 *Assuming Specialty Palliative Care is available in your setting...*

 **How likely is it that you would request input from Specialty Palliative Care to help manage the patient's goals of care in this scenario?**

- Not at all likely (1)
- A little bit likely (2)
- Somewhat likely (3)
- Very likely (4)

Q20 **How would you prefer to interact with Specialty Palliative Care in this scenario?**
 (Mark all that apply.)

- Care Process Model (1)
- Telephone conversation (2)
- E-consult (specialist provider reviews the case electronically and provides recommendations on management in a dedicated note in the electronic record) (3)
- Traveling Specialist (4)
- Integrated Community Specialist (e.g. primary/specialty collaboration with options of collocated visits, e-consults or curbsides) (5)
- Virtual consults (6)
- Curbside (e.g., call a known colleague for advice) (7)
- Email (8)
- Reviewing Specialty Palliative Care notes in the EHR (9)
- Other (10) ________________________________________________

|  |
| --- |

|  |
| --- |

Q21 **Managing Goals of Care** Mr. Smith is an 85-year-old man with history of diabetes mellitus, congestive heart failure, and chronic obstructive pulmonary disease who has been living in an independent senior apartment with his wife who serves as the primary caregiver.  He was admitted to the ICU yesterday for sepsis secondary to pneumonia.  He has required intubation and vasopressor support and now shows evidence of renal failure that may require the addition of dialysis.  He does not have an advanced directive on file.  When you talk to his wife about goals of care, she becomes tearful and fearful.  She notes that they have never talked about what types of medical interventions he may want.  She asks you for support as she tries to understand the medical complexities, prognosis, and options. 

 **On a scale of 0 to 10, where 0 is "Not at all confident" and 10 is "Completely confident", how confident are you in your ability to manage the goals of care on your own?**

- Not at all confident 0 (0)
- 1 (1)
- 2 (2)
- 3 (3)
- 4 (4)
- 5 (5)
- 6 (6)
- 7 (7)
- 8 (8)
- 9 (9)
- Completely confident 10 (10)

Q22 **What resources or specialties would you request input from to help manage the goals of care in this scenario?** (Mark all that apply.)

- ⊗None (1)
- Volunteers trained in Advanced Care Planning (2)
- Chaplain (3)
- Ethics consult (4)
- Hospice (5)
- Psychiatry (6)
- Social worker (7)
- Specialty Palliative Care (8)
- Other (9) ________________________________________________

Q23 **How likely is it that you would request input from Specialty Palliative Care to help manage the goals of care in this scenario?**

- Not at all likely (1)
- A little bit likely (2)
- Somewhat likely (3)
- Very likely (4)

Q24 **How would you prefer to interact with Specialty Palliative Care in this scenario?**
 (Mark all that apply.)

- Care Process Model (1)
- Telephone conversation (2)
- E-consult (specialist provider reviews the case electronically and provides recommendations on management in a dedicated note in the electronic record) (3)
- Traveling Specialist (4)
- Integrated Community Specialist (e.g. primary/specialty collaboration with options of collocated visits, e-consults or curbsides) (5)
- Virtual consults (6)
- Curbside (e.g., call a known colleague for advice) (7)
- Email (8)
- Reviewing Specialty Palliative Care notes in the EHR (9)
- Other (10) ________________________________________________

Q25 **About You**

**How long have you been in practice?**

_______ years (1)

Q26 **In what clinical area or specialty do you mainly practice?**

- Emergency Room (1)
- Hospital (Hospitalist) (2)
- Primary Care (3)
- Specialty (please specify) (4) ________________________________________________
- Other (5) ________________________________________________

Q27 **What is your best estimate of the total percent of your patients who are in need of palliative care?**

|  | 0 | 10 | 20 | 30 | 40 | 50 | 60 | 70 | 80 | 90 | 100 |
| --- | --- | --- | --- | --- | --- | --- | --- | --- | --- | --- | --- |

| Percent (1) | 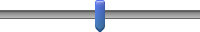 |
| --- | --- |

Q28 **Have you received formal training in palliative care (e.g., palliative care fellowship, dedicated palliative care CME or training activities)?**

- Yes (1)
- No (2)

Q29 **Would you like additional training in palliative care?**

- Yes (1)
- No (2)

Q30 **Would you like to receive training in palliative care?**

- Yes (1)
- No (2)

Q31 **Prior to this survey, were you aware of the Palliative Care Consulting Service?**

- Yes (1)
- No (2)

Q32 **How many times have you used the Palliative Care Consulting Service?**

- 0 Never (1)
- 1-5 times (2)
- 6-10 times (3)
- More than 10 times (4)

Q33 THANK YOU FOR COMPLETING THE SURVEY!
  **Please click SUBMIT to record your answers.**
