# Supplementary material for: Physicians’ Confidence in Primary Palliative Care and Preferred Methods of Responding: A Sequential Mixed-Methods Survey
Source: Palliat Med Rep. 2025 Apr 29;6(1):215–22. doi: 10.1089/pmr.2024.0110 (PMC12410319; doi:10.1089/pmr.2024.0110)
Supplement: Supplementary Appendix [file pmr.2024.0110_supplementaryappendix.docx]

**Appendix.** Clinical Vignettes

| **Topic** | **Vignette** |
| --- | --- |
| Family dynamics | Mrs. Smith is an 83-year-old woman who has been hospitalized for 25 days after the development of a neurologic condition known as posterior reversible encephalopathy syndrome. She is comatose, and the consensus of the primary and neurology consulting teams is that she will not improve enough to survive this hospitalization. She does not have an advance directive and her daughters are in conflict as to what their mother’s wishes would be. On multiple occasions, when you talk with the daughters, the conversations are very tense and do not reach consensus. |
| Symptoms | Mr. Jones is a 54-year-old man with widely metastatic rectal carcinoma with diffuse peritoneal cancer involvement. He is losing his ability to maintain his oral hydration and nutrition, and his nausea has been refractory to the medications on the nausea order set. He and his wife are struggling with how to proceed and are hoping his nausea can improve. |
| Pain | Mr. Dolor is a 72-year-old man with metastatic prostate cancer with persistent and severe pain that interferes with his function despite escalating doses of opioid pain medications. A recent consultation with interventional pain colleagues resulted in interventions providing only temporary relief. You are hoping to manage his pain medically. |
| Emotional and spiritual distress | Mr. Pickle is a 42-year-old man with advanced heart failure who has had repeated and prolonged hospitalizations. On morning rounds, he expresses his wish to have his life ended. He expresses ongoing frustration with his poor quality of life and just wants to “get this over with.” Mr. Pickle is very sick but not necessarily dying. He is in extreme emotional distress. |
| Goals of care - patient | Mrs. Anderson is an 83-year-old woman who you have been caring for over the course of many years but has begun to show signs of mild cognitive impairment. She has been quite independent over the years and has had strong opinions regarding her medical care. At present she retains the capacity for decision-making and expressing her goals. You note that a conversation on advanced-care planning and goals of care is needed before she loses her ability to express her desires. |
| Goals of care - spouse | Mr. Smith is an 85-year-old man with a history of diabetes, congestive heart failure, and chronic obstructive pulmonary disease who has been living in an independent senior apartment with his wife who serves as the primary caregiver. He was admitted to the intensive care unit yesterday for sepsis secondary to pneumonia. He has required intubation and vasopressor support and now shows evidence of kidney failure that may require the addition of dialysis. He does not have an advance directive on file. When you talk to his wife about goals of care, she becomes tearful and fearful. She notes that they have never talked about what types of medical interventions he may want. She asks you for support as she tries to understand the medical complexities, prognosis, and options. |
